# Alveoli‐Like Multifunctional Scaffolds for Optical and Electrochemical In Situ Monitoring of Cellular Responses from Type II Pneumocytes

**DOI:** 10.1002/advs.202301395

**Published:** 2023-05-28

**Authors:** Seonghyeon Eom, So Yeon Lee, Jung Tae Park, Inhee Choi

**Affiliations:** ^1^ Department of Life Science University of Seoul Seoul 02504 Republic of Korea; ^2^ Department of Chemical Engineering Konkuk University Seoul 05029 Republic of Korea; ^3^ Department of Applied Chemistry University of Seoul Seoul 02504 Republic of Korea

**Keywords:** alveoli‐like scaffolds, electrochemical monitoring, gold nanoparticles, metal–organic framework, optical monitoring

## Abstract

While breathing, alveoli are exposed to external irritants, which contribute to the pathogenesis of lung disease. Therefore, in situ monitoring of alveolar responses to stimuli of toxicants under in vivo environments is important to understand lung disease. For this purpose, 3D cell cultures are recently employed for examining cellular responses of pulmonary systems exposed to irritants; however, most of them have used ex situ assays requiring cell lysis and fluorescent labeling. Here, an alveoli‐like multifunctional scaffold is demonstrated for optical and electrochemical monitoring of cellular responses of pneumocytes. Porous foam with dimensions like the alveoli structure is used as a backbone for the scaffold, wherein electroactive metal–organic framework crystals, optically active gold nanoparticles, and biocompatible hyaluronic acid are integrated. The fabricated multifunctional scaffold allows for label‐free detection and real‐time monitoring of oxidative stress released in pneumocytes under toxic‐conditions via redox‐active amperometry and nanospectroscopy. Moreover, cellular behavior can be statistically classified based on fingerprint Raman signals collected from the cells on the scaffold. The developed scaffold is expected to serve as a promising platform to investigate cellular responses and disease pathogenesis, owing to its versatility in monitoring electrical and optical signals from cells in situ in the 3D microenvironments.

## Graphical abstract

A novel scaffold is demonstrated for label‐free biomarker detection and real‐time monitoring of cellular behaviors. It contains electroactive metal–organic frameworks and optically active gold nanoparticles, providing multiple sensing modes for in situ monitoring of cellular responses. This offers a novel approach for studying alveolar responses to toxicants and a multifunctional cellular scaffold for exploring cellular behavior under 3D microenvironments.

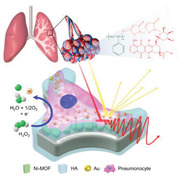

## 1 Introduction

The alveoli are the main units involved in respiration and are normally filled with air upon inhalation. Due to this, the alveoli are easily exposed to potentially hazardous materials and air pollution. Alveolar diseases are caused by the inhalation of noxious chemical components, such as cigarette smoke,^[^ ^1^ ^]^ humidifier disinfectants,^[^ ^2^ ^]^ and airborne microplastics.^[^ ^3^ ^]^ As lower respiratory tract infections, chronic obstructive pulmonary disease, lung cancer, and tuberculosis are among the ten most common causes of death worldwide.^[^ ^4^ ^]^ Therefore, understanding the human lung and its alveolar responses to potentially toxic components is important. To monitor cellular responses under complex and dynamic environments mimicking in vivo living systems,^[^ ^5^, ^6^ ^]^ 3D cell culture models, such as cellular scaffolds,^[^ ^7^ ^]^ spheroids,^[^ ^8^ ^]^ organoids,^[^ ^9^ ^]^ and organ on a chip^[^ ^10^ ^]^ have recently drawn much attention. Due to the technological advances in fabrication methods, accurately reproducing the 3D cellular microenvironment has been successfully achieved by assembling cells to their designed shape; however, most of them used conventional bioassays of cellular lysates and fluorescent labeling for measuring cellular responses after culturing. Among them, cellular scaffolds have great advantages in concurrently integrating functions for both 3D cell cultures and real‐time monitoring of cellular responses.^[^ ^11^, ^12^ ^]^ In order to directly monitor responses from the 3D cell cultures, it is crucial to insert sensing modalities onto the scaffolds, resembling the structure and environment of in vivo systems. For this purpose, several materials, carbon frameworks,^[^ ^13^, ^14^ ^]^ plasmonic composites,^[^ ^15^, ^16^, ^17^, ^18^ ^]^ and polymer composites^[^ ^22^, ^23^, ^24^ ^]^ have been introduced for fabricating 3D scaffolds with superior optical and electrical properties and proper porosity, enabling efficient mass transfer.

Metal–organic frameworks (MOFs), known as crystalline materials, are composed of regular chemical bonds between metal ions and organic ligands.^[^ ^22^ ^]^ Depending on their components, the MOFs possess various chemical structures, unique morphologies, large surface areas, tunable pore distributions, and sufficient redox sites.^[^ ^23^ ^]^ Owing to these intrinsic properties, MOFs have recently found application in diverse areas, such as in energy storage and conversion devices,^[^ ^22^ ^]^ gas separation membranes,^[^ ^24^ ^]^ drug delivery carriers,^[^ ^25^ ^]^ and sensing materials.^[^ ^26^ ^]^ The active metal sites and porosity of the MOF‐derived materials are very profitable in creating electrochemical and optical sensing layers.^[^ ^26^ ^]^ For instance, redox‐active MOF‐derived structures, including CoFe‐Prussian blue analogues composites^[^ ^27^ ^]^ and Co‐MOF/titanium nanosheet array^[^ ^28^ ^]^ were served as sensing layers for electrochemically detecting biomolecules, such as hydrogen peroxide and glucose.

Gold nanoparticles (AuNPs) play an important role as versatile biosensing probes due to their excellent optical and electrical properties, easy surface modification, and biocompatibility.^[^ ^29^ ^]^ AuNPs have unique and tunable optical and electrical features that vary with their shape and size,^[^ ^30^, ^31^, ^32^ ^]^ and their diverse combinations with other materials are extremely promising in biomedical fields, including molecular diagnosis,^[^ ^33^ ^]^ drug and gene delivery,^[^ ^34^, ^35^ ^]^ cancer treatment,^[^ ^36^ ^]^ and tissue engineering.^[^ ^37^, ^38^ ^]^ In particular, they effectively amplify optical signals, such as Raman scattering and Rayleigh scattering due to electromagnetic field enhancement by localized surface plasmon resonance (LSPR) effect.^[^ ^30^, ^39^ ^]^ Alongside optical properties, the high electrical conductivity of the AuNPs allows them to function as electrodes for electrochemical sensors. For example, the gold nanostructure‐integrated conductive microwell arrays have been used for electrochemically monitoring glioblastoma spheroids.^[^ ^40^, ^41^ ^]^

**Figure 1:**
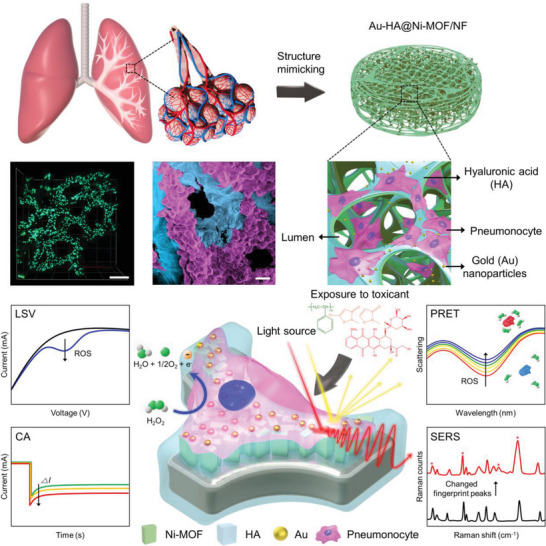
Overall schematic illustrations of cellular response multifunctional monitoring using alveoli‐like 3D hollow MOF‐based scaffolds (lower center). Fabrication of Au‐HA@Ni‐MOF scaffolds that mimic alveolar morphology, and fluorescence images, and electron microscope images of cells cultured on the scaffold are shown (upper). Cell scaffolds were exposed to three toxicants, followed by electrochemical and optical monitoring using four multi‐mode detection modalities (lower).

Here, we suggest a multifunctional 3D alveoli‐like scaffold (called Au‐HA@Ni‐MOF/NF) for real‐time monitoring of cellular responses based on both electrochemical and optical signals. In order to mimic the alveolar structure, nickel foam (NF) is used as a backbone of the scaffold where electroactive MOF crystals and optically active plasmonic AuNPs are integrated via simple fabrication procedures, as described in **Figure** **1**. Owing to the electrochemical and optical sensing modalities introduced on the 3D scaffold, cellular responses, such as the generation of reactive oxygen species and the secretion of cellular molecules can be monitored by multiple means through amperometry and nanospectroscopy providing label‐free electrical signals (e.g., linear‐sweep voltammetry, LSV; chronoamperometry, CA) and optical signals (e.g., plasmon resonance energy transfer, PRET; surface‐enhanced Raman scattering, SERS). Our alveoli‐like 3D cellular scaffold, therefore, provides versatile platforms for investigating cellular responses upon exposure to drugs and environmental toxicants. We describe below a series of detailed characteristics and monitoring principles.

## 2 Results and Discussion

### 2.1 Structural and Compositional Properties of 3D Cellular Scaffold

**Figure 2:**
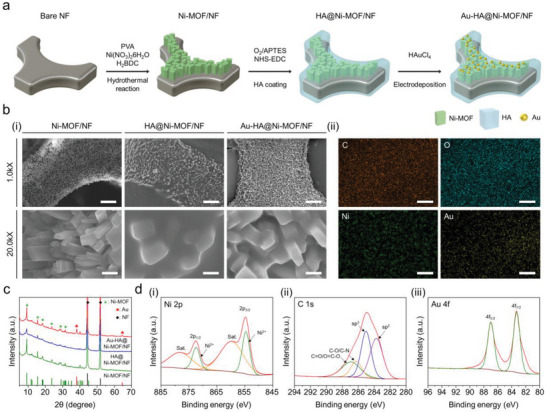
Structural and compositional properties of Au‐HA@Ni‐MOF/NF. a) Schematic illustration of the fabrication of Au‐HA@Ni‐MOF/NF. b) Scanning electron micrographs of the fabricated structures. i) FE‐SEM images of Ni‐MOF/NF, HA@Ni‐MOF/NF, and Au‐HA@Ni‐MOF/NF. Scale bars are 20 µm (upper) and 1 µm (lower). ii) Elemental mapping images of Au‐HA@Ni‐MOF/NF (scale bar: 1 µm) performed using FE‐SEM. c) XRD patterns of the prepared samples, simulated NF (black line, JCPDS No. 04‐0850), Ni‐MOF (green line, JCPDS No. 035‐1677), and Au (red line, JCPDS No. 04‐0784). d) High‐resolution XPS spectra of Au‐HA@Ni‐MOF/NF: Ni 2*p* (i), C 1*s* (ii), and Au 4*f* (iii).

As illustrated in **Figure** **2**a, the Au‐HA@Ni‐MOF was prepared through a three‐step synthetic process. First, Ni‐MOF was directly grown on a nickel foam (NF) through a facile hydrothermal reaction, using nickel nitrate hexahydrate as the metal precursor and terephthalic acid as the organic linker. In this process, NF served as a self‐sacrificial template to afford nickel ions where Ni‐MOF nanosheets can be vertically grown.^[^ ^42^ ^]^ Furthermore, nickel cations and terephthalate anions, derived from the metal precursor and organic linker, respectively, were linked to form Ni‐MOF on the surficial nickel of NF (Figure S1, Supporting Information).^[^ ^43^ ^]^ After the hydrothermal process, biocompatible hyaluronic acid (HA) was coated on Ni‐MOF/NF to increase cell viability. In the final step, AuNPs were coated on the HA‐Ni‐MOF/NF by electrodeposition in a HAuCl_4_ solution through the following reduction Equation 1 (Figure S1, Supporting Information):^[^ ^44^, ^45^ ^]^

(1) $${\mathrm{AuCl}}_{4}^{-}+3e^{-}\to \mathrm{Au}\left(0\right)+{\mathrm{Cl}}^{-}$$

Digital photographs of Ni‐MOF/NF, HA@Ni‐MOF/NF, and Au‐HA@Ni‐MOF/NF show that all final products were homogeneously grown on NF (Figure S2, Supporting Information). Electron micrographs in Figure 2b(i) show changes in morphologies according to each synthesis step of the Au‐HA@Ni‐MOF/NF. The Ni‐MOF/NF exhibits nanocuboids with a width of 200–300 nm and a length of 700–800 nm grown on the NF framework, which provides a much higher surface area (3.26 m^2^ g^−1^) than the bare NF (0.29 m^2^ g^−1^) (Figure S3, Supporting Information). After the HA coating process, a smooth layer was formed over the surface of the structure. In Figure 2b(ii), energy dispersive spectrometer (EDS) mapping images clearly reveal that the representative elements constituting the structure, such as C, O, Ni, and Au, are homogeneously distributed on the surface of Au‐HA@Ni‐MOF/NF, which was further validated using other structural and compositional analyses. In Figure 2c, the X‐ray diffraction (XRD) patterns of all samples exhibit the distinct diffraction peaks at 2*θ* = 44.48° and 51.8° indexed to (1 1 1) and (2 0 0) crystal lattices of the NF substrate.^[^ ^46^ ^]^ The diffraction peaks at 9.2°, 11.8°, 15.6°, 18.3°, 18.6°, and 23.8° observed in all samples contributed to (2 0 0), (0 1 0), (1 0 1), (1 1 0), and (0 2 0) planes of the Ni‐MOF.^[^ ^47^ ^]^ Furthermore, the unit cell structure of Ni‐MOF ([Ni_3_(OH)_2_(1,4‐BDC)_2_(H_2_O)_4_]⋅2H_2_O)^[^ ^48^ ^]^ is constructed with infinite layers parallel to the (0 1 0) planes and each layer has 1D chains composed of nickel octahedra and terephthalic acid (Figure S4, Supporting Information).^[^ ^49^ ^]^ These results indicate the existence of Ni‐MOF and NF in all samples. In particular, the peaks at 38.2° and 64.6° for the Au‐HA@Ni‐MOF/NF corresponded to the (1 1 1) and (2 2 0) planes in the general crystal lattice of gold.^[^ ^50^ ^]^ Moreover, field‐emission transmission electron microscope (FE‐TEM) analysis was conducted to confirm the crystalline structure and morphologies of Ni‐MOF and AuNPs, detached from the NF by sonication (Figure S5a, Supporting Information). As shown in Figure S5b (Supporting Information), Ni‐MOF exhibits a nanosheet structure with angular edges and a width of 500 nm, which corresponds to the field‐emission scanning electron microscope (FE‐SEM) images (Figure 2b). Furthermore, according to Bragg's law,^[^ ^51^ ^]^ the resolved inter‐planar distance of 0.96 nm is attributed to *d*‐spacing of (2 0 0) crystal plane of Ni‐MOF, which is consistent with the XRD results. Meanwhile, the angular edges partially disappeared, and the width was increased in the case of Au‐HA@Ni‐MOF (Figure S5c, Supporting Information), implying the coating of HA polymer on the surface of Ni‐MOF. Furthermore, spherical AuNPs with a radius of 20 nm were observed on the surface of Au‐HA@Ni‐MOF. In particular, the interplanar distance of 0.23 nm in the high magnification FE‐TEM image corresponds to the (1 1 1) crystal plane of Au, consistent with the peak of 38.2° in the XRD pattern (Figure 2c).^[^ ^52^ ^]^ The elemental mapping images in Figure S5d and Table S1 (Supporting Information) show that AuNPs are successfully deposited on the surface of HA@Ni‐MOF. The chemical structures of the samples were explored using Fourier‐transform infrared spectroscopy (FT‐IR) (see Figure S6, Supporting Information) to probe the suggested synthetic procedure (Figure S1, Supporting Information). According to the IR spectra of Ni‐MOF/NF, the peaks at 3608 and 1499 cm^−1^ were observed due to the stretching vibrations of a hydroxyl group (—OH) and para‐aromatic group, respectively. Moreover, Ni‐MOF/NF showed characteristic peaks at 1569 and 1373 cm^−1^, corresponding to the asymmetric and symmetric stretching vibrations of the carboxylate group (—COO—) in terephthalate anions, respectively.^[^ ^53^ ^]^ These results indicate that the MOF was successfully constructed with H_2_BDC and Ni(NO_3_)_2_⋅6H_2_O, which acted as an organic linker and metal precursor. Meanwhile, the characteristic peaks at 1035 and 1619 cm^−1^ were attributed to the C—O—C and carboxylate asymmetric stretching modes, implying that HA was coated on Ni‐MOF/NF.^[^ ^54^, ^55^ ^]^ To further verify the elemental composition and chemical states of the Au‐HA@Ni‐MOF/NF, X‐ray photoelectron spectroscopy (XPS) was used. The XPS survey spectra and estimated atomic percent of elements (see Figure S7a and Table S2, Supporting Information) revealed that the Au‐HA@Ni‐MOF/NF is composed of Ni, Au, C, O, and N. Ni, C, and O exist as fundamental components of Ni‐MOF, and particularly, C and O are excessively distributed due to the coating of HA. In addition, it can be observed that a small amount of N and Si are also distributed owing to the 3‐aminopropyltriethoxysilane (APTES) pre‐treatment in the coating process of HA (see Figure S7, Supporting Information). High‐resolution XPS analysis was performed to confirm the chemical state of each element. In particular, Ni 2*p* spectra shown in Figure 2d(i) revealed the chemical state of Ni ions that existed in Ni‐MOF. In detail, Ni 2*p* spectra exhibited two major satellite peaks (denoted as “Sat.”) of Ni 2*p*_3/2_ and Ni 2*p*_1/2_ at 859.7 and 878.1 eV, respectively. Furthermore, the Ni 2*p* peaks located at 854.6 and 872.4 eV were assigned to Ni^2+^ 2*p*_3/2_ and Ni^2+^ 2*p*_1/2_, serving as active sites for electrochemical reactions.^[^ ^28^, ^56^, ^57^ ^]^ The Au 4*f* XPS spectra exhibited two significant peaks at 83.3 and 87.0 eV corresponding to the Au 4*f*_7/2_ and 4*f*_5/2_ characteristic signals, respectively.^[^ ^58^ ^]^ These results indicate that Au was successfully electrodeposited on the surface of HA@Ni‐MOF/NF with the support of hydroxyl and amino groups of HA, which anchor to stabilize Au ions in the HAuCl_4_ solution.^[^ ^59^ ^]^ The C 1*s* spectra in Figure 2d(ii) revealed four peaks at binding energies of 287.4, 286.4, 285.0, and 283.7 eV, attributed to C—O/O=C—O, C—O/C—N, *sp* ^3^, and *sp* ^2^ bonds, respectively.^[^ ^60^, ^61^ ^]^ In Figure 2d(iii), the peak, placed at the binding energy of 530.0 eV, corresponds to oxygen atoms combined with metal (Ni). The other peaks at 531.2 and 532.1 eV in the O 1*s* spectra (Figure S7b, Supporting Information) represent the C=O/O—H bond and C—O groups, respectively.^[^ ^62^ ^]^ These peaks in C 1*s* and O 1*s* spectra are due to the bonds in terephthalic acid (i.e., a ligand in Ni‐MOF), and HA.

### 2.2 Optical Properties of the 3D Cellular Scaffolds

**Figure 3:**
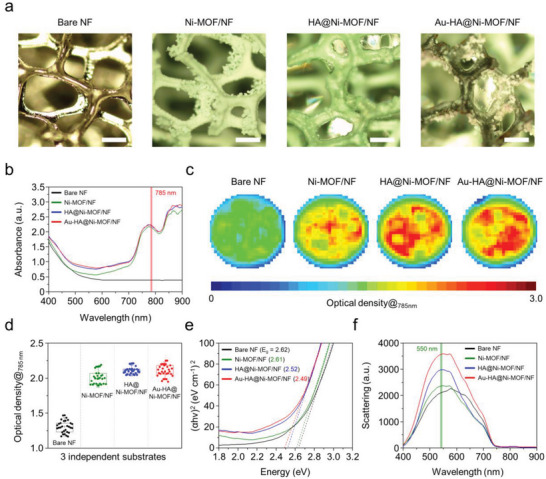
Optical properties of Au‐HA@Ni‐MOF/NF and its derivatives a) Optical micrographs (scale bar: 200 µm). b) Spectral absorbance profile. c) UV–vis endpoint mapping images for optical density at 785 nm. d) Optical profile of 10 pixels representing the highest intensity in endpoint mapping images (*n* = 3). e) Optical energy band gap tauc plot. f) Hyperspectral scattering profiles.

In **Figure** **3**a, optical micrographs exhibit color and morphological changes in accordance with each step for fabricating the final Au‐HA@Ni‐MOF/NF. After modification of NF with MOF nanocuboids, the structure turned greenish and roughened from the intrinsically smooth NF surface. Following HA grafting, the surface of the structures became smooth again. After depositing AuNPs onto the structure, brownish agglomerates of the particles along with the MOF nanocuboids were finally observed. Absorbance spectra exhibit a broad range of absorption peaks from visible to near‐infrared (NIR) wavelengths, as shown in Figure 3b after the sequential coating process of the NF. Importantly, emerging peaks at the NIR region are profitable to use, longer wavelength light sources (e.g., 785 nm laser) for SERS without damaging cells and biomolecules.^[^ ^63^, ^64^ ^]^ In Figure 3c, mapping images of optical density at 785 nm show that the developed scaffold (i.e., Au‐HA@Ni‐MOF/NF) can evenly absorb the 785 nm light for SERS signal enhancement. The box plots shown in Figure 3d indicate that the optical density at 785 nm for the modified scaffolds is 1.6 times higher than the initial bare NF structure. A change in the onset of absorption corresponds to a change in the band gap,^[^ ^65^ ^]^ which was determined from a tauc plot (see Figure 3e). The tauc plot method is given by the following equation:

(2) $${\left(\alpha hv\;\right)}^{2}=\;k\left(hv-E_{g}\right)$$

Where “*α*” is the absorption coefficient, “*h*” is the Planck constant, “*v*” is the photon's frequency, “*k*” is an energy conversion constant, and “*E*_g_” is the band gap energy. The valence band energies of bare NF and Au‐HA@Ni‐MOF/NF were 2.62 and 2.49 eV, respectively. The decrease in the band gap suggests that the absorption of photons by Au‐HA@Ni‐MOF/NF was greater over a wider range of wavelengths. The lower band gap can be a crucial contributor to improve electrical conductivity and optical performance.^[^ ^65^ ^]^ The scattering spectra of Au‐HA@Ni‐MOF/NF and its derivatives were examined using a dark‐field microscope equipped with a spectro‐photometer (see Figure 3f). Taking the opacity of the substrate into account, the scattering spectra of the surface were measured using the reflectance mode of the microscope. As a result, *λ*_max_ were determined to be 577 and 550 nm and FWHMs were 208 and 157 nm, for the bare NF and the Au‐HA@Ni‐MOF/NF, respectively (see Figure S8, Supporting Information). To take advantage of the cytochrome *c* (Cyt *c*)‐mediated PRET for further ROS monitoring, a specific scattering peak ≈550 nm based on absorption for Cyt *c* is essentially required.^[^ ^66^ ^]^ Therefore, 3D scaffolds were prepared for further label‐free optical monitoring based on PRET and fingerprint Raman signals through the sequential modification steps.

### 2.3 Type II Pneumocyte Cultured on the Alveoli‐Like Scaffolds

**Figure 4:**
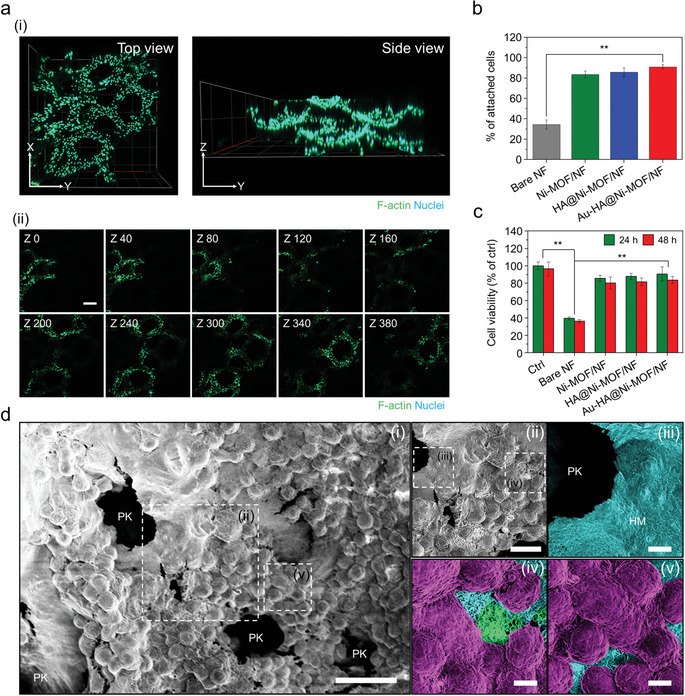
3D evaluation of cellular adhesion and viability on the scaffold. a) 3D confocal imaging of the cells. i) Top and side view immuno‐fluorescence images of the cells cultured on the Au‐HA@Ni‐MOF/NF. ii) *Z*‐stack images (stack interval: 1 µm, scale bar: 200 µm). b) Cellular adhesion efficiency on the scaffolds. c) Cell viability of the cells cultured on the scaffolds evaluated by CCK‐8 assay. d) SEM images of the alveoli‐like hollow 3D scaffold. i) Low magnification image of alveolar mimicking 3D scaffold (500×, scale bar: 40 µm). ii) Magnified sectional image (1,000×, scale bar: 20 µm). iii–v) Magnified and color‐coded sectional images showing pore of Kohn (PK), HA membrane (iii, cyan) (3,000×, scale bar: 5 µm) and the cells (purple) attached to the scaffolds. (Scale bar: 5 µm)

Using the fabricated scaffolds, cellular adhesion and viability on their structures were first tested. The A549 cell line, known as type II pneumocytes, was selected to monitor cellular behaviors since these cells produce and secrete pulmonary surfactants, which are considered precursors to type I pneumocytes. It has been previously reported that the A549 cell line exhibits ultrastructural, metabolic, and transport properties consistent with type II pneumocytes in vivo.^[^ ^67^, ^68^, ^69^, ^70^ ^]^ In order to analyze the cellular distribution on the scaffold, immunofluorescence imaging of cytoskeleton and nucleus was performed. Confocal images confirm that the cultured cells are distributed along the backbone of the scaffold 3D (see **Figure** **4**a(i)). In Figure 4a(ii), *z*‐stack images with an interval of 1 µm distinctly show the even distribution of cells through the scaffold. Additionally, the 3D scanning image allows the nuclei to be distinguished with higher resolution, and the scaffold structure covered with cells was observed (Movie S1, Supporting Information). In Figure 4b, the formula for the calculated cell adhesion efficiency is Equation 2:

(3) $$\mathrm{Cell}\;\mathrm{adhesion}\;\mathrm{efficiency}\;\left(\%\right)=\frac{\mathrm{Total}\;\mathrm{seeding}\;\mathrm{cell}\;\mathrm{number}-\mathrm{Remaining}\;\mathrm{cell}\;\mathrm{number}}{\mathrm{Total}\;\mathrm{seeding}\;\mathrm{cell}\;\mathrm{number}}\;\times 100$$

The adhesion efficiency increases according to the serial surface modification steps, which reached 90.9% for the finally modified Au‐HA@Ni‐MOF/NF, which is 2.6 times enhanced adhesion compared with the bare NF. Furthermore, the viability of cells cultured on the modified NF scaffolds for 48 h increased by ≈2.2–2.3 times compared with bare NF, suggesting that the modified scaffolds are quite stable under the 3D growth conditions compared with other substrates (see Figure 4c). After a week of long‐term culture, Au‐HA@Ni‐MOF/NF exhibited excellent cell viability indicating the stability of 3D expanded culture areas and the biocompatibility of the scaffold (see Figure S9, Supporting Information). The morphological similarity with the native alveoli was further observed using scanning electron micrographs (see Figure 4d). The human alveoli have pores, such as the alveolar lumen and pores of Kohn (PK) with diameters of 200–300 and 3–20 µm, respectively,^[^ ^71^ ^]^ which are very similar with the pore sizes of fabricated scaffolds (see Figure S10, Supporting Information).^[^ ^72^ ^]^ In Figure 4d, it is clearly observed that cells were tightly attached onto the scaffold. Moreover, the HA membrane spread thinly between the NF pores resembles a blood‐alveoli membrane in the alveoli sac (see Figure 4d(i–iii) and Movie S2, Supporting Information). Through the color‐coded high‐magnification image for better visibility, the characteristic morphology of the round A549 cells (purple) and HA membrane (HM, cyan) coated on the scaffold were observed (see Figure 4d(iv,v)). To identify the structural features of the scaffold, the scaffold where cells were partially removed from the NF framework, with pores similar to the size of the alveoli lumen (LM), was imaged (see Figure S11(i), Supporting Information). The high‐magnification image shows the distribution of all components of the fabricated scaffold along with the lumen space and NF backbone (see Figure S11(ii), Supporting Information). In Figure S11(iii) (Supporting Information), the existence of MOF fragment structure for electrical conduction on the scaffold was observed. Interestingly, in the high‐magnification distribution, most of the cells tightly adhered to the HA membrane (see Figure 4d(v)), which suggests that this is a result of interaction with the RGD peptide of the cell or the extracellular matrix. It also provides a more stable environment by forming a network with the polar head of surfactant phospholipids secreted from type II pneumocytes.^[^ ^73^, ^74^ ^]^

### 2.4 Electrochemical Monitoring of ROS Generated from Cells on the Alveoli‐Like Scaffold

**Figure 5:**
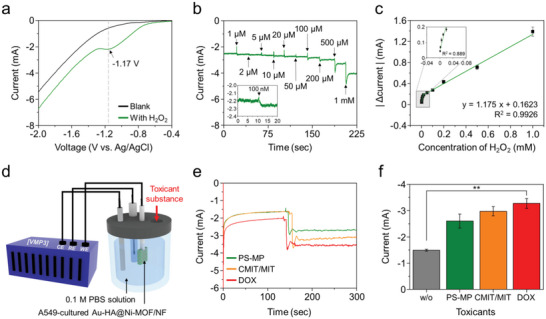
Electrical monitoring of ROS released from cells on the Au‐HA@Ni‐MOF/NF. a) LSV of Ni‐MOF/NF with/without 10 mM H_2_O_2_ in 0.1 m PBS. b) Amperometric transient current density versus time plot of Au‐HA@Ni‐MOF/NF under H_2_O_2_ injection of different concentrations from 100 nM to 1 mM. c) Calibration curve of H_2_O_2_ concentration versus |Δ current|. d) Schematic illustration of the electrochemical ROS monitoring set‐up from the A549 cell‐cultured on Au‐HA@Ni‐MOF/NF in a three‐electrode configuration. e) Plots for the amperometric transient current densities at an applied potential of −1.17 V versus Ag/AgCl versus time plot after cell culture on the Au‐HA@Ni‐MOF/NF with an injection of different toxicant of PS‐MPs, CMIT/MIT, and DOX. f) Comparison of current densities before and after stimulation with different toxicants.

Prior to cellular monitoring, we first checked the electrochemical behavior of Au‐HA@Ni‐MOF/NF toward H_2_O_2_, the LSV was performed directly using the Ni‐MOF/NF as a working electrode with a geometric surface of 1.0 cm^2^. As shown in **Figure** **5**a, Ni‐MOF/NF exhibited a distinct reduction peak at ≈−1.17 V versus Ag/AgCl when measured in 0.1 m phosphate buffered saline (PBS) solution with 10 mM H_2_O_2_, due to the reduction reaction of H_2_O_2_ with Ni^2+^ at the specific potential.^[^ ^56^ ^]^ In particular, the distinct reduction peak of Ni‐MOF/NF implies a superior sensing activity compared to other scaffold materials, such as bare NF and FeNiLDH/NF (Figure S12a, Supporting Information). It should be noted that the 3D porous architecture of the scaffold and large surface area of Ni‐MOF offers excellent accessibility and quickly facilitates the transport of electrons and ions.^[^ ^75^ ^]^ The electrochemical sensing performance of Au‐HA@Ni‐MOF/NF for H_2_O_2_ was evaluated through the CA at the applied potential of −1.17 V versus Ag/AgCl. As presented in Figure 5b, the cathodic current rapidly decreased and stayed at a steady state with the addition of H_2_O_2_ at different concentrations from 1 µM to 1 mM, and a low detection limit was calculated as 100 nM according to a signal‐to‐noise factor. The calibration curve, derived from the CA measurement, shows two linear response ranges for H_2_O_2_, 0.001 to 0.02 mM (low concentrations) and 0.02 to 1 mM (high concentrations). The appearance of two linear ranges in the calibration curve can be attributed to the nature of electrochemical detection showing relatively high sensitivity at low concentrations and interference among reactants and products at high concentrations by inner‐sphere electrode reaction mechanism.^[^ ^76^ ^]^ Even in high concentration region, the calibration curve of Au‐HA@Ni‐MOF/NF presented an excellent sensitivity of 1175 µA cm^−2^ mM^−1^ with a good linear relationship (*R* ^2^ = 0.9926), which is much higher than the other scaffolds, such as HA/NF (43 µA cm^−2^ mM^−1^) and HA@FeNiLDH/NF (73 µA cm^−2^ mM^−1^). The superior detection performance of Au‐HA@Ni‐MOF/NF is due to the large surface area of Ni‐MOF and abundance of active nickel ions in electrochemical reduction reaction.^[^ ^56^, ^77^, ^78^ ^]^ Encouraged by this performance of Ni‐MOF for H_2_O_2_ sensing, ROS generated from the cells cultured on the alveolar‐mimicking scaffold (i.e., Au‐HA@Ni‐MOF/NF) was monitored. Figure 5d shows the electrochemical monitoring set‐up to measure the current change from the A549 cultured scaffold upon exposing toxicants. From the CA results (Figure 5e), sudden current drop and its maintenance thereafter were observed according to the toxicants, such as air bone polystyrene microplastics (PS‐MPs), 5‐chloro‐2‐methylisothiazol‐3(2H)‐one (CMIT/MIT), and doxorubicin (DOX). Based on the calibration curve and current changes before and after the exposure of the toxicant to the cells on the scaffold (see Figure 5f), the amount of cellular ROS production in the case of PS‐MPs exposure was estimated to be comparable with 18 µM H_2_O_2_. Notably, the current increment due to ROS released from the cells exposed to DOX and CMIT/MIT was ≈4.7 and 3.2 times higher than the case of PS‐MPs that was well consistent with the following optical measurement of ROS. In addition, through real‐time detection of electrochemical signals from the cells exposed to toxicants for 1 h, the stability of Au‐HA@Ni‐MOF/NF was verified (Figure S13, Supporting Information).

### 2.5 Optical Monitoring of ROS Generated from Cells on the Alveoli‐Like Scaffold

**Figure 6:**
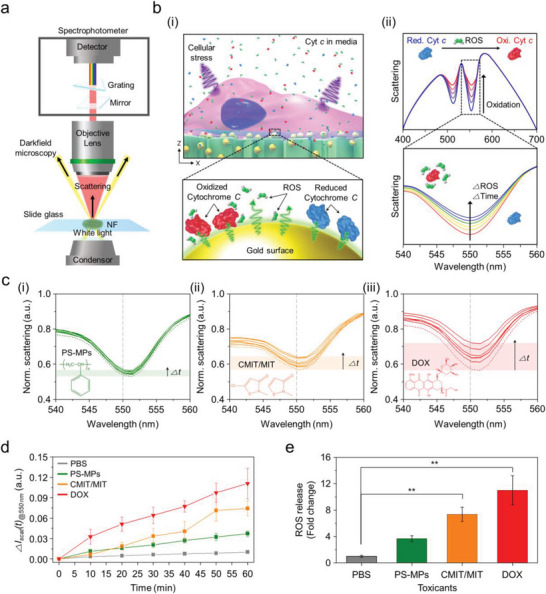
Optical monitoring of oxidative stress responses to toxicants in A549 cells via the Cyt *c*‐mediated PRET effect. a) Configuration of hyperspectral monitoring set‐up combined with darkfield microscopy. b) Schematic description of optical ROS monitoring using PRET effect. i) Detail of Cyt *c*‐mediated PRET effect on ROS release on cellular scaffolds. ii) Expected changes in spectral quenching dip induced by Cyt *c*‐mediated PRET as a response to ROS release. c) Time‐resolved PRET spectra according to toxicants, i) PS‐MPs, ii) CMIT/MIT, and iii) DOX. d) Changes in intensity at 550 nm wavelength for the tested toxicants. e) Plot for fold changes for ROS release from A549 cells after 1 h exposure to toxicants.

Next, we applied the proposed alveoli‐like scaffold for optically monitoring the cellular responses exposed to toxicants, which was conducted using dark‐field nanospectroscopy (see **Figure** **6**a). To monitor ROS levels in real‐time, PRET effect of redox‐active Cyt *c*, measured with molecular‐specific quenching dips,^[^ ^66^, ^82^ ^]^ was employed. In brief, PRET is a phenomenon in which a unique quenching dip that corresponds to the molecule absorption wavelengths appears in the Rayleigh scattering spectrum of plasmonic nanoparticles when the light‐absorbing molecules are in close proximity to the surface. To measure the change in quenching dip, redox‐active Cyt *c* was applied as a model for a light‐absorbing molecule and used Cyt *c*‐mediated PRET (see Figure 6b(i,ii)). Two spectral quenching dips exactly matched with absorption wavelengths at 520 and 550 nm of the reduced Cyt *c*, and these quenching dips change with the oxidized state of Cyt *c*. Owing to the optimized scattering properties of the he fabricated Au‐HA@Ni‐MOF/NF (see Figure 3f), it shows clear spectral quenching dips of Cyt *c* absorption band, compared with other scaffolds (i.e. HA@FeNiLDH/NF and HA/NF), and probes (i.e., AuNPs only) additionally tested for the detection performance (see Figure S14, Supporting Information). The detection limit of our scaffold was also found to be 1.12 nM, which was superior to other tested substances (Figure S15, Supporting Information). Thus, we finally monitored the oxidative stress upon exposure to PS‐MP, CMIT/MIT, and DOX from A549 cells cultured on the Au‐HA@Ni‐MOF/NF. In Figure 6d,e, when the increments were calculated and plotted by fold change, ROS increased by 3.7 and 7.4 times for PS‐MPs and CMIT/MIT compared with the control (i.e., PBS), respectively, and the highest result was ≈11 times for cells exposed to DOX. This result is consistent with the trend of the current density change of A549 cells by toxicants in Figure 5f, which clearly demonstrates an increase in the relative ROS level compared to untreated A549 cells, indicating a response to external stimulation in A549 cells. After 60 min, the extracellular ROS levels were measured in a range comparable to 1 µM^−1^ mM H_2_O_2_ for all three conditions based on the estimation from the calibration curve. This result can be interpreted that oxidative stress is significantly induced in the tested exposure conditions, based on the previous study reporting that extracellular hydrogen peroxide induces apoptosis when >0.7 µM.^[^ ^83^ ^]^ Taken together, this further suggests that responses to the external stimuli of A549 cells can also be optically monitored on the proposed cellular scaffold.

### 2.6 Label‐Free Raman Analysis for Cellular Responses on the Alveoli‐Like Scaffold

**Figure 7:**
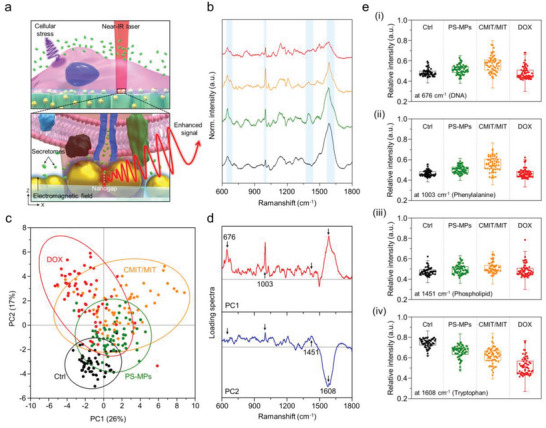
In situ detection of Raman signals from cells cultured on the scaffold. a) Schematic illustration of SERS detection of cellular response on the scaffold. b) Representative Raman spectra of cells cultured on the Au‐HA@Ni‐MOF/NF. c) PCA score scatter plot for the cells treated with different toxicants. d) Loading spectra at PC1 and PC2. e) Quantitative box plot for Raman intensity at i) 676 cm^−1^, ii) 1003 cm^−1^, iii) 1451 cm^−1^, and iv) 1608 cm^−1^ measured from the A549 cells exposed to different toxicants (triplicate, *n* = 20).

Raman spectroscopy is a representative label‐free optical technique that enables non‐destructive monitoring of cells and their secretomes.^[^ ^84^ ^]^ AuNPs were deposited on the scaffold to form an electromagnetic field (EM) for SERS (see Figure 3b).^[^ ^85^ ^]^ The schematic illustration in **Figure** **7**a shows the enhancement of the Raman signal by the EM nanogap formed between the AuNPs and MOF crystals. Specific Raman signals of molecules secreted from cells exposed to each toxicant were displayed with representative spectra (see Figure 7b). In these spectra, changes by toxicants in the assigned cell‐specific peaks could be observed at a glance. Each spectrum exhibits heterogeneous peaks due to complex components (e.g., nucleic acids, proteins, lipids, and polysaccharides) on the cellular surface, the Raman peaks assignment is contained in Table S3 (Supporting Information). Therefore, statistically significant peaks of each spectrum can be identified through principal component analysis (PCA).^[^ ^86^, ^87^ ^]^ PCA is a statistical analysis that computes key patterns and uses them to perform a simplification of a data set. In Figure 7c, the PCA scores using PC1 and PC2 were plotted to distinguish the cellular responses under different toxicant conditions, which were found to capture 43% of the overall variance. The scatter plot distributions of PC1 and PC2 for each condition clearly reveal a distinct clustering pattern in which the formed ellipse represents a 95% confidence interval. In Figure 7d, PCA loading plots of PC1 and PC2 exhibit prominent Raman peaks which contribute significantly to the discrimination. The characteristic peak intensity at 676 cm^−1^ belonging to DNA bases peak (e.g., guanine and cytosine) decreased only in the DOX group (see Figure 7e(i)), and the peak intensity at 1003 cm^−1^ attributed to the protein peak (e.g., phenylalanine) increased in PS‐MPs and CMIT/MIT (see Figure 7e(ii)). In Figure 7e(iii), the vibrational peaks for phospholipids and amide II (1451 cm^−1^) in all exposure groups showed no significant alterations and unstable signal differences were observed. However, the peak intensity at 1608 cm^−1^ attributed to the tryptophan and amide group, consistently decreased in all treated conditions (see Figure 7e(iv)). In situ Raman detection in live cells is very useful in monitoring dynamic changes in complex cellular components unlike the static data obtained from dead cells. It has previously been reported that Raman spectra possess complex information for changes in cellular components and thus their spectral patterns dynamically change according to the apoptosis in each phase.^[^ ^88^, ^89^, ^90^ ^]^ For example, the decrease at 676 cm^−1^ (i.e., DNA) and increase in intensity at 1003 cm^−1^ (i.e., phenylalanine) in the spectrum of the DOX‐treated group reflect the late phase of apoptosis where nuclear fragments are expelled from the cells. The increased DNA‐related Raman intensity in the PS‐MPs‐treated group may be related with chromatin condensation and nuclear fragmentation as part of the apoptotic process. However, the differences in phospholipid compositions of all groups are tiny, so it is difficult to explain the changes in CMIT/MIT‐treated group only with apoptosis. Eva et al. reported that changes in the corresponding Raman signal patterns can classify cellular toxicity into necrosis and apoptosis.^[^ ^90^ ^]^ Among the toxicants, CMIT/MIT‐treated group exhibited considerable changes in Raman intensities at protein related peaks (increased at 1003 and decreased at 1608 cm^−1^), which are closely involved in cellular necrosis. Raman signal pattern observed in Figure 7e indicates that necrosis and apoptosis can be triggered by CMIT/MIT, which is supported by other reports.^[^ ^91^, ^92^ ^]^ Taken together, this result suggests that the interpretation of Raman spectra monitored on the proposed cellular scaffolds enables us to classify the cellular toxic behaviors.

## 3 Conclusion

The Ni‐MOF with nanoblock morphology was first prepared on the surface of alveoli‐mimicking NF by a facile hydrothermal reaction using nickel precursor and terephthalic acid. Subsequently, the HA and AuNPs were coated and deposited to improve the biocompatibility and optical sensing properties of the scaffold, respectively. For real‐time monitoring of the cellular response exposed to potentially noxious components, the A549 cells were cultured on the fabricated alveoli‐like scaffolds. The developed platform allows to electrochemically monitor oxidative stress in living cells on the scaffold, owing to its excellent electrical properties. Moreover, cellular oxidative stress could be also monitored in real‐time via redox‐active Cyt *c*‐mediated PRET signal and cellular behaviors could be statistically classified based on the Raman spectral patterns obtained from the treated cells. The developed multifunctional scaffold has the potential to be applied in various fields through scalability enabled by excellent mechanical properties, ease of modification and integration to other platforms (e.g., microfluidic devices), which can provide a new strategy for monitoring cellular behavior under 3D culture conditions.

## 4 Experimental Section

### Materials and Chemicals

The following chemicals were obtained from Sigma–Aldrich (St. Louis, MO, USA): Ni(NO_3_)_2_∙6H_2_O (98.5%), H_2_BDC (98%), polyvinyl alcohol (PVA) (Mw = 89,000–98,000, 99+% hydrolyzed), and HAuCl_4_∙3H_2_O (≥ 99.9%). Ethanol and *N, N*‐dimethylformamide (DMF) were purchased from Duksan Chemicals (Ansan, Korea). Nickel foam (NF, ≥ 99.5%) and hydrogen peroxide (H_2_O_2_, 35 wt.%, stab) were purchased from Invisible Inc. (Suwon, Korea) and Alfa Aesar (Seoul, Korea), respectively. Deionized (DI) water was obtained and used through the purifying system to 6.8 pH and 18 Ω cm^−1^. 3‐aminopropyltriethoxysilane (APTES), gold(iii) chloride trihydrate (HAuCl_4_), glutaraldehyde (70%), MES hydrate (99.5%), cytochrome *c* from equine heart (Cyt *c*), L‐Ascorbic acid (AA), phosphate buffered saline (PBS), and Hoechst 33258 were purchased from Sigma–Aldrich (St. Louis, MO, USA). 5‐chloro‐2‐methylisothiazol‐3(2H)‐one (CMIT/MIT), 1‐ethyl‐3‐(3‐dimethylaminopropyl) carbodiimide (EDC), *N*‐Hydroxysulfosuccinimide (Sulfo‐NHS), Alexa Fluor 488 phalloidin (F‐actin), Penicillin/streptomycin (PenStrep), and Trypsin‐EDTA (0.05%) were purchased from Thermo Fisher Scientific (Waltham, MA, USA). RPMI 1640 medium was purchased from Lonza Biosciences (Morrisville, NC, USA). Fetal bovine serum (FBS) was purchased from MP Biomedicals (Irvine, CA, USA). Cell Counting Kit‐8 (CCK‐8) was purchased from Dojindo Laboratories (Kumamoto, Japan). Paraformaldehyde (PFA) was purchased from Biosesang (Seongnam, Korea). Polydimethylsiloxane (PDMS, Sylgard 184) elastomer kit was purchased from Dow Corning (Midland, MI, USA). Ethanol (99.9%) was purchased from Samchun Chemical Co. (Seoul, Korea). Polystyrene microplastics (PS‐MPs) were purchased from Polysciences, Inc. (Warrington, PA, USA). Doxorubicin Hydrochloride salt (DOX) was purchased from LC Laboratories (Woburn, MA, USA). Hyaluronic acid (HA) was obtained from Hyundai Bioland (Cheongju, Korea). All chemicals and solvents were obtained from commercial sources and used without further purification.

### Synthesis of Ni‐MOF on NF

First, NF pieces (25×75 mm) were washed by sonication in HCl solution and ethanol for 30 min one after the other, to eliminate impurities, such as oxides on the surface. Ni(NO_3_)_2_∙6H_2_O (0.72 g) and PVA (0.132 g) were dissolved in DI water (20 mL) to make a clear solution. Furthermore, a green solution was made by dissolving H_2_BDC (0.42 g) in DMF (40 mL). After sufficient stirring, the prepared homogeneous solutions were mixed and transferred into a 100 mL Teflon‐lined stainless steel autoclave with the washed NF. Then, it was heated and maintained in an oven at 130°C for 24 h, and naturally cooled to ambient temperature. The obtained Ni‐MOF/NF was washed with DI water and ethanol several times and dried in a vacuum oven at 50°C overnight.

### Surface Modification of HA@Ni‐MOF/NF

The prepared Ni‐MOF/NF was treated with O_2_ plasma (CUTE‐1MPR‐Dual Mode, Femto science, Hwaseong, Korea) under the following conditions: 100 w/flow rate 40 sccm/5×10^−2^ Torr, for 3 min on both the front and back sides. Then, Ni‐MOF/NF was loaded in 5% APTES solution and stirred at 200 rpm for 2 h at 60°C, and were washed three times with PBS to remove unbound APTES and dried in an oven at 65°C for ≈1 h. Next, 10 mM EDC and 10 mM Sulfo‐NHS were mixed together in a 50 mM MES buffer solution containing 0.5% HA (MW: 1.3 to 1.8 MDa). The mixed solution was evenly placed on the amination of Ni‐MOF/NF and incubated overnight at room temperature (RT) under a vacuum under reduced pressure. Finally, the residual HA mixture was washed three times with PBS and then completely dried at RT before use in the next step.

### Fabrication of Au‐HA@Ni‐MOF/NF

AuNPs were deposited on the as‐prepared surface of HA@Ni‐MOF/NF via the electrodeposition method, which was conducted in a three‐electrode configuration with HA@Ni‐MOF, a Pt coil, and Ag/AgCl (1 m KCl) as the working, counter, and reference electrode, respectively. HAuCl_4_ dissolved in DI water (4 mM, 20 mL) was used as the electrolyte. The electrodeposition was performed at an applied potential of −0.2 V (vs. Ag/AgCl) for 300 s at RT. Finally, the product (Au‐HA@Ni‐MOF/NF) was washed with ethanol several times and dried at RT overnight.

### Structural and Compositional Characterizations of the Fabricated Scaffolds

The morphology and chemical composition of the products grown on NF was observed and analyzed using field emission scanning microscopy (FE‐SEM, Hitachi SU 8010, Hitachi High‐Tech, Tokyo, Japan) and energy‐dispersive X‐ray spectroscopy (EDS) at an acceleration voltage of 10 kV. The high‐resolution X‐ray diffraction (HR‐XRD, SmartLab, Rigaku, Tokyo, Japan) analysis in the 2‐theta range of 5 to 70°at a scan rate of 2.5° min^−1^ using Cu K*α* radiation (*λ* = 1.5418 Å) was used to identify the crystalline structures of Ni‐MOF and AuNPs. Field‐emission transmission electron microscopy (FE‐TEM, JEM‐F200, JEOL Ltd, Akishima, Japan) equipped with an EDS system (JEOL Dual SDD type, 100 mm^2^) operating at an acceleration voltage of 200 kV was used to identify the nanosheet morphologies and crystalline structures of Ni‐MOF and Au‐HA@Ni‐MOF. Furthermore, the specific chemical structures of Ni‐MOF and HA were confirmed by Fourier transform infrared (FT‐IR, Spectrum two, Perkin Elmer, Waltham, MA, USA) spectroscopy in the spectral range of 500 to 4000 cm^−1^. The chemical composition and chemical states of elements in the scaffolds were confirmed by energy‐dispersive X‐ray spectroscopy (EDS) and X‐ray photoelectron spectroscopy (XPS, K‐alpha, Thermo Fisher Scientific, Waltham, MA, USA), respectively.

### Optical Characterizations of Au‐HA@Ni‐MOF/NF

Using a dark‐field microscope (BX 43, Olympus, Tokyo, Japan), low‐magnification images (5×, NA 0.15) of Au‐HA@Ni‐MOF/NF were obtained. Reflection spectra extracted from 40 randomly chosen pixels from the high‐magnification (20×, NA 0.45) images were collected using a microscope equipped with hyperspectral imaging spectrophotometer (Cytoviva, Auburn, AL, USA). The absorbance spectrum and optical density distribution were mapped over using the well‐scan mode over the entire area of Au‐HA@Ni‐MOF/NF and its derivatives (96 well size, 6 mm diameter) using a multi‐mode microplate reader (SpectraMax iD3, Molecular Devices, San Jose, CA, USA). The direct optical bandgap was determined by tauc plotting using each absorbance spectrum value. The tauc plot method was measured using Equation 2. Values of the tangent line and the intercept of the tauc plot were determined by utilizing linear fitting in OriginPro (v9.0, OriginLab Corporation, Northampton, MA, USA).

### Cell Culture

Human type II pneumocyte (A549) cells were purchased from the Korean Cell Line Bank (KCLB) and cultured according to the standard protocols. The cells were cultured in RPMI 1640 medium supplanted with 10% FBS and 1% PenStrep at 37°C in a 5% CO_2_ humidified incubator. The number of cells was counted using an automatic cell counter (ADAM‐MC, NanoEntek, Seoul, Korea) to determine the seeding density. A549 cells (8×10^4^ cells well^−1^) were seeded on the scaffold and incubated at overnight.

### Cell Attachment Test

The cells were cultured on the scaffolds for 7 h, immersed in a complete medium and then gently transferred to another plate. Subsequently, all residual media and cells were centrifuged at 1000 rpm for 3 min, then resuspended and counted by ADAM‐MC. The cellular adhesion efficiency was calculated using Equation 3.

### Cell Viability of Type II Pneumocyte on the Scaffolds

Cell viability test was performed using CCK(WST)‐8 assay. After culturing cells on the scaffold for a duration of 1–7 days, 20 µL of a reagent was added to 200 µL of medium. After 2 h, only the reagent was carefully transferred to a new 96‐well plate. Subsequently, it was determined as endpoint mode at 450 nm wavelength using a microplate reader and then tested three times as a biological repeat for each condition. During culture, replacement of the growth medium was performed on the day following the initial seeding, and then every two days thereafter.

### Immunofluorescence Imaging

The large‐area distribution of the cells cultured on the scaffold was observed by using a confocal laser scanning microscope (LSM 800, Carl Zeiss NTS Ltd., Jena, Germany). After 48 h incubation of A549 cells on the scaffolds, the cells were fixed with 4% PFA and washed three times with PBS. For staining the cytoskeleton inside the cells, Alexa Fluor 488 phalloidin (1:250) was used and the reaction lasted for 1 h at RT, and then the unbound dyes were washed three times with PBS. Counterstaining to indicate the location of the cells was performed using nuclear dye Hoechst 33258 (1:400). To observe cell distribution, *Z*‐stack images were acquired up to 400 µm depth using a *z*‐stack spacing of 1 µm. 3D analysis and processing were performed with Zen 3.3 software (Zeiss, Jena, Germany).

### FE‐SEM Characterization of the Cell Adhered on the Scaffold

After 48 h incubation of cells on the scaffold, it was immersed in a 4% glutaraldehyde solution and allowed to react overnight at 4°C. Then, the gradient dehydration was applied with different concentrations of ethanol (from 10% to 99.9%, increasing by 10%, 10 min for each step) and dried in the air. The samples were placed on a stub and images were captured using an FE‐SEM after Pt sputtering for 120 s at 17 mA. The SEM images were processed and color‐coded for additional information and better visualization of locations.

### Electrochemical Detection of Cellular Oxidative Stress

All the electrochemical measurements were conducted in a typical three‐electrode configuration using an impedance/electrical analyzer (VMP3, Bio‐Logic, Grenoble, France) electrochemical workstation at RT. To identify the performance of the Ni‐MOF as an electrochemical H_2_O_2_ sensor, the Ni‐MOF/NF (1×1×0.5 cm), platinum coil, and Ag/AgCl (saturated with KCl) were employed as working electrode, counter electrode, and reference electrode, respectively. The supporting electrolyte was 0.1 M PBS solution. LSV was performed at a scan rate of 2 mV s^−1^ with and without the H_2_O_2_ solution. The process of detecting H_2_O_2_ was performed using CA at a constant voltage of −1.17 V (vs. Ag/AgCl) with the injection of H_2_O_2_ solution. The real‐time electrochemical monitoring of cellular responses was conducted using the A549‐cultured Au‐HA@Ni‐MOF/NF as the working electrode with the injection of toxicant agents, such as PS‐MPs, CMIT/MIT, and DOX.

### Optical Detection of Cellular Oxidative Stress via PRET

For PRET‐based ROS monitoring, 200 µM Cyt *c* was reduced by 100 mM AA for 1 h for use as a redox probe (5:1 v/v). Toxicants, such as PS‐MPs, CMIT/MIT, and DOX were adjusted to treat the final concentration when mixed with the pre‐reduced Cyt *c* solution, based on previous reports.^[^ ^93^, ^94^, ^95^ ^]^ The changes in scattering spectra induced by the oxidation reaction between Cyt *c* and ROS from A549 on cellular scaffolds were monitored using a dark field microscope combined with a hyperspectral spectrophotometer. A 20× reflective objective lens (NA 0.45) was used for imaging. The integration time for collecting the spectra was 0.2 s.

### Confocal Raman Measurement of Cellular Responses

The cellular responses of A549 cells after exposure to each toxicant were measured using a confocal micro‐Raman spectrometer (LabRAM HR Evolution, HORIBA Scientific, Kyoto, Japan). For Raman measurement, a 785 nm high power diode laser was focused on the A549 cells through 50× long working distance objective lens (NA 0.5). The laser power was dynamically controlled with an ND filter, and the backscattered light was collected through the same objective lens used for excitation and transferred to a spectrometer (600 gr mm^−1^ grating). Raman spectra of each condition were collected with acquisition time 3 s and accumulation three times. PCA for all Raman spectra was performed using the Unscrambler X software (CAMO software, Oslo, Norway). Before the PCA, the spectra were made smooth using a Savitsky–Golay filter, pretreatments, such as baseline correction and normalization were applied to each raw SERS spectrum. The data was mean‐centered and a full cross‐validation PCA was performed using two PCs at 600 to 1800 cm^−1^ range.

### Statistical Analysis

All data were obtained after the experiments were repeated at least thrice. The data presented were the mean ± standard error of mean (S.E.M.) for each experiment. The statistical significance was assessed via one‐way ANOVA followed by the Tukey HSD post hoc analysis (*p < 0.05, ^**^ *p* < 0.01, and ^***^ *p* < 0.005, compared to control) using SPSS software (v25.0, IBM, NY, USA).

## Conflict of Interest

The authors declare no conflict of interest.

## Supporting information

Supporting Information

Supplemental Movie 1

Supplemental Movie 2

## Data Availability

The data that support the findings of this study are available from the corresponding author upon reasonable request.
