# Supplementary material for: Alveoli‐Like Multifunctional Scaffolds for Optical and Electrochemical In Situ Monitoring of Cellular Responses from Type II Pneumocytes
Source: Adv Sci (Weinh). 2023 May 28;10(23):2301395. doi: 10.1002/advs.202301395 (PMC10427368; doi:10.1002/advs.202301395)
Supplement: Supplementary file 1 — Supporting Information [file ADVS-10-2301395-s001.pdf]

ADVANCED SCIENCE
Open Access

Supporting Information

for *Adv. Sci.*, DOI 10.1002/advs.202301395

Alveoli-Like Multifunctional Scaffolds for Optical and Electrochemical In Situ Monitoring of Cellular Responses from Type II Pneumocytes

Seonghyeon Eom, So Yeon Lee, Jung Tae Park and Inhee Choi**

Supporting Information

Alveoli-like Multifunctional Scaffolds for Optical and Electrochemical in-situ Monitoring of Cellular Responses from Type II Pneumocytes

Seonghyeon Eom, So Yeon Lee, Jung Tae Park, and Inhee Choi**

S. Eom, Prof. I. Choi
Department of Life Science, University of Seoul, Seoul, 02504, Republic of Korea
E-mail: inheechoi1@uos.ac.kr

S.Y. Lee, Prof. J.T. Park
Department of Chemical Engineering, Konkuk University, Seoul, 05029, Republic of Korea
E-mail: jtpark25@konkuk.ac.kr

Prof. I. Choi
Department of Applied Chemistry, University of Seoul, Seoul 02504, Republic of Korea

Table of Contents

List of abbreviations and their descriptions used in this manuscript S4

Table S1. Atomic percent of element (at%) obtained from EDS mapping images of Au-HA@Ni-MOF/NF (corresponding to Figure 2b(ii)) S5

Table S2. Atomic percent of element (at%) obtained from XPS spectra of Au-HA@Ni-MOF/NF S6

Table S3. Raman peak assignments references list table (corresponding to Figure 7(b))....... S7

Figure S1. Schematic illustration of chemical structures during synthetic procedure of Ni-MOF growth on the NF, sequential HA coating, and gold electrodeposition processes. S8

Figure S2. Digital photographs of samples according to each synthesis step of the Au-HA@Ni-MOF/NF S9

Figure S3. N_2 adsorption/desorption isotherm of Bare NF and Ni-MOF/NF S10

Figure S4. Illustration of the unit cell of Ni-MOF structure (CCDC No. 638866). Gray, brown, red, and pink balls represent Ni^{2+} ions, carbon, oxygen, and hydrogen atoms, respectively. S11

Figure S5. Structural and compositional properties of Ni-MOF and Au-HA@Ni-MOF S12

Figure S6. FT-IR spectra of the synthesized NF, Ni-MOF/NF, HA@Ni-MOF/NF, and Au-HA@Ni-MOF/NF S13

Figure S7. Surface chemical composition analysis of Au-HA@Ni-MOF/NF...................... S14

Figure S8. Approximating Gaussian distribution using each scattering data. The exhibition for full width at half maximum (FWHM) and maximum peak (λ_{max}) in a Gaussian distribution S15

Figure S9. Assessment of long-term (1 week) cell viability on Au-HA@Ni-MOF evaluated by CCK-8 assay.. S16

Figure S10. Low magnification FE-SEM images of the 3D structure of bare Ni foam........ S17

Figure S11. SEM images of the alveoli-like hollow 3D scaffold. S18

Figure S12. Comparison of electrochemical performance of the scaffolds. S19

Figure S13. Stability of the electrochemical detection: chronoamperometry test of A549 cell-cultured Au-HA@Ni-MOF/NF S20

Figure S14. Cyt *c* selectivity test of Au-HA@Ni-MOF/NF and other comparative substrates S21

Figure S15. Quantification of H_2O_2 through controlled performance comparison experiments of the substrates S22

List of abbreviations and their descriptions used in this manuscript.

Abbreviations	Full form/Description
NF	Nickel Foam
Ni-MOF	Nickel – Metal-Organic Framework grown on NF
HA@Ni-MOF	Hyaluronic acid coated Ni-MOF/NF
Au-HA@Ni-MOF	Au deposited HA@Ni-MOF/NF

Table S1. Atomic percent of element (at%) obtained from EDS mapping images of Au-HA@Ni-MOF/NF (corresponding to **Figure 2b(ii)**).

Ni	Au	C	O	N	Total
5.74 %	9.01 %	36.06 %	38.05 %	11.14 %	100 %

Table S2. Atomic percent of element (at%) obtained from XPS spectra of Au-HA@Ni-MOF/NF.

Ni	Au	C	O	N	Si	Total
1.61 %	3.58 %	53.09 %	27.75 %	7.53 %	6.44 %	100 %

Table S3. Raman peak assignments references list table (corresponding to **Figure 7(b)**).

Peak (cm^{-1})	Assignment
676	DNA/RNA
782	Ring stretching of cytosine and thymine
788	O−P−O stretching of DNA phosphodiester bonds
1003	Ring stretching of phenylalanine
1013	C−O stretching of deoxyribose and other sugars
1033	C−H in phenylalanine
1049	C−O in deoxyribose
1066	DNA back bone, C−C in lipids
1080	
1128	C−C in lipids; C−N in proteins
1209	C−C_6H_5 stretching of phenylalanine
1323	Amide Ⅲ alpha helix
1451	CH_2, CH_3 deformation, phospholipids
1482	Amide Ⅱ
1572	G, A (Guanine, Adenine)
1608	C=C tyrosine, tryptophan
1650	Amide Ⅰ

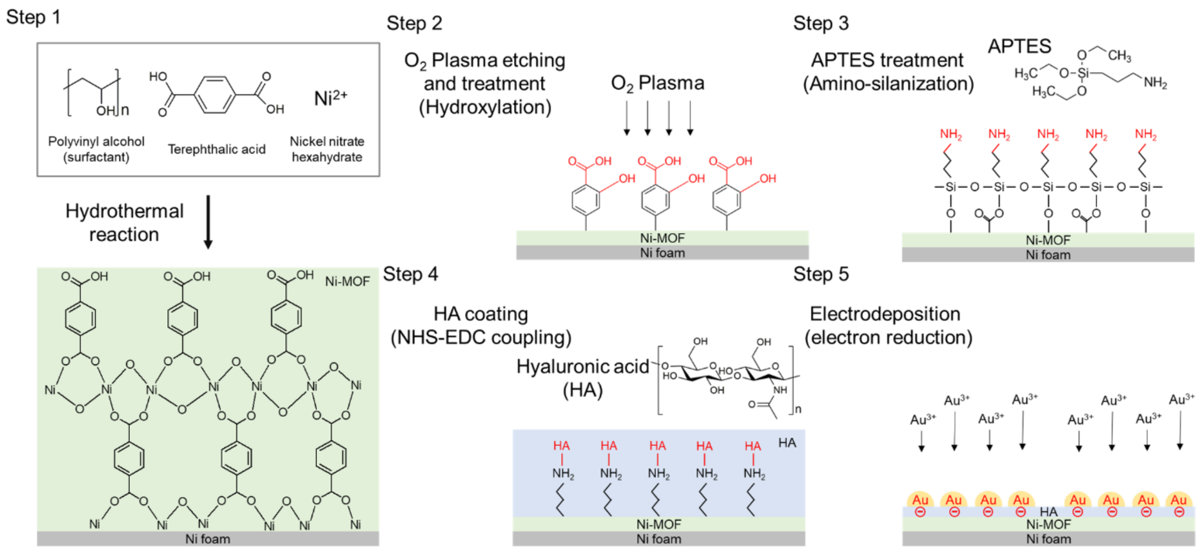

Figure S1. Schematic illustration of chemical structures during synthetic procedure of Ni-MOF growth on the NF, sequential HA coating, and gold electrodeposition processes.

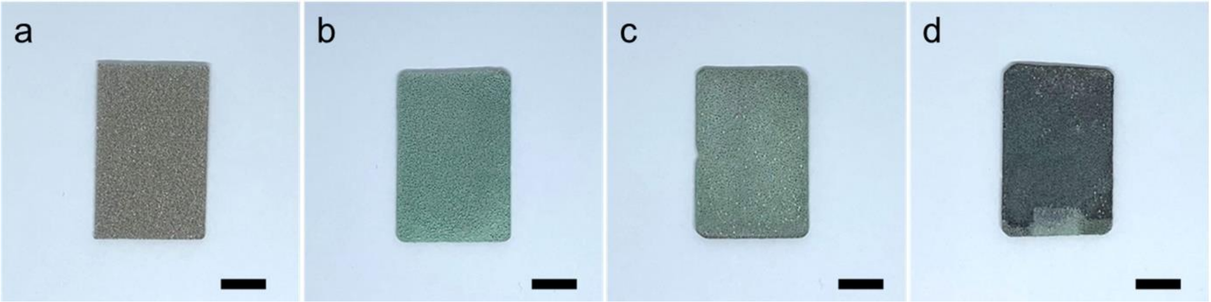

Figure S2. Digital photographs of samples according to each synthesis step of the Au-HA@Ni-MOF/NF: (a) Bare NF, (b) Ni-MOF/NF, (c) HA@Ni-MOF/NF, and (d) Au-HA@Ni-MOF/NF (scale bar: 10 mm).

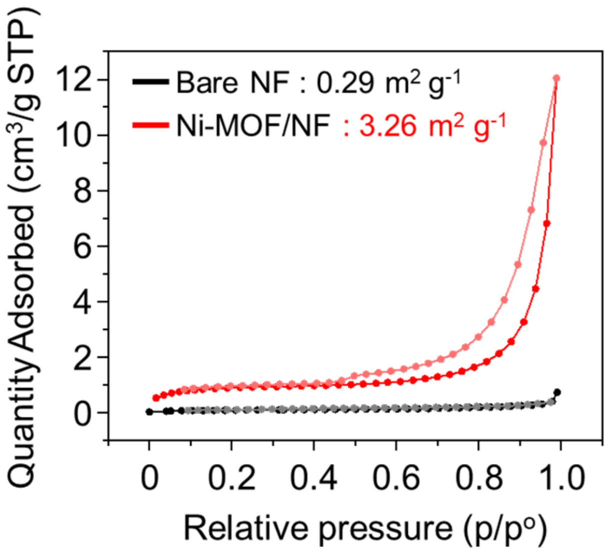

Figure S3. N_2 adsorption/desorption isotherm of Bare NF and Ni-MOF/NF.

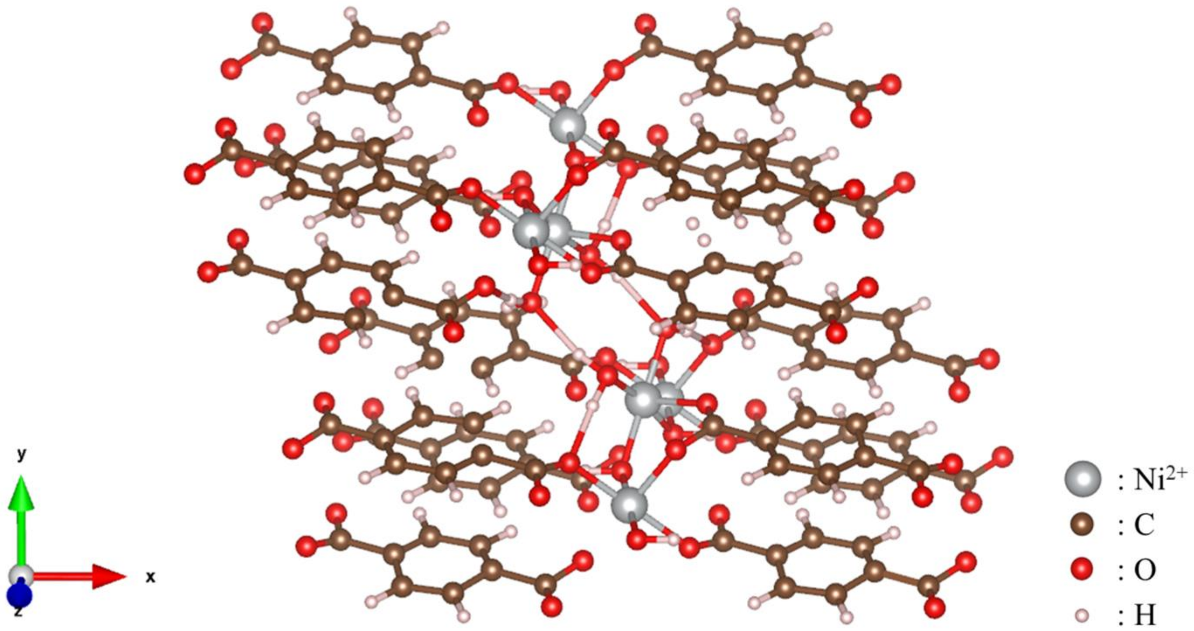

Figure S4. Illustration of the unit cell of Ni-MOF structure (CCDC No. 638866). Gray, brown, red, and pink balls represent Ni^{2+} ions, carbon, oxygen, and hydrogen atoms, respectively.

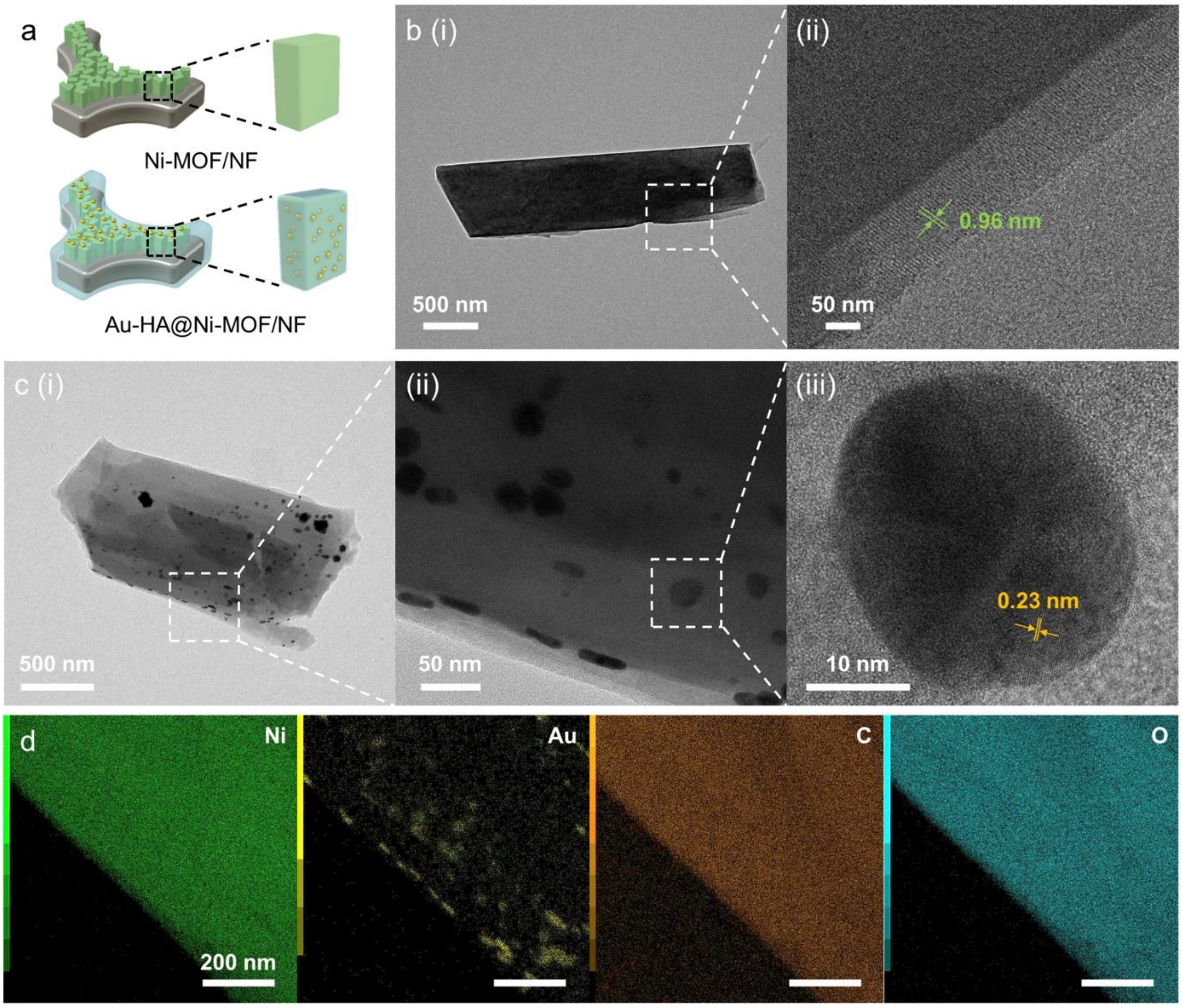

Figure S5. Structural and compositional properties of Ni-MOF and Au-HA@Ni-MOF. (a) Schematic illustration of Ni-MOF and Au-HA@Ni-MOF particles detached from the NF backbones via ultrasonication. (b, c) FE-TEM images of Ni-MOF (b) and Au-HA@Ni-MOF (c) particles under different magnifications. (b-ii) and (c-iii) show high-magnification images with an interlayer spacing of Ni-MOF and AuNPs. (d) Elemental mapping images of the Au-HA@Ni-MOF particle characterized using FE-TEM.

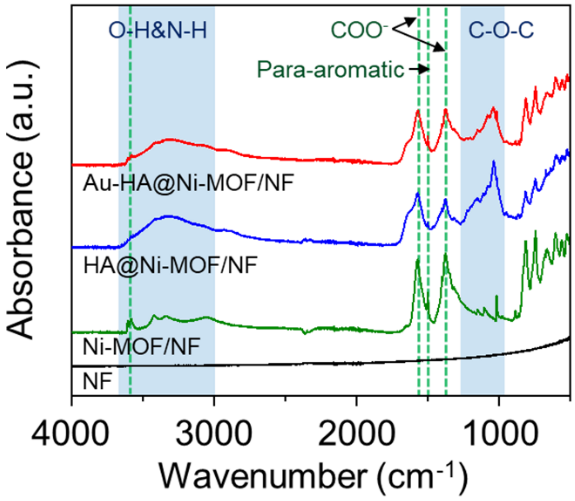

Figure S6. FT-IR spectra of the synthesized NF, Ni-MOF/NF, HA@Ni-MOF/NF, and Au-HA@Ni-MOF/NF.

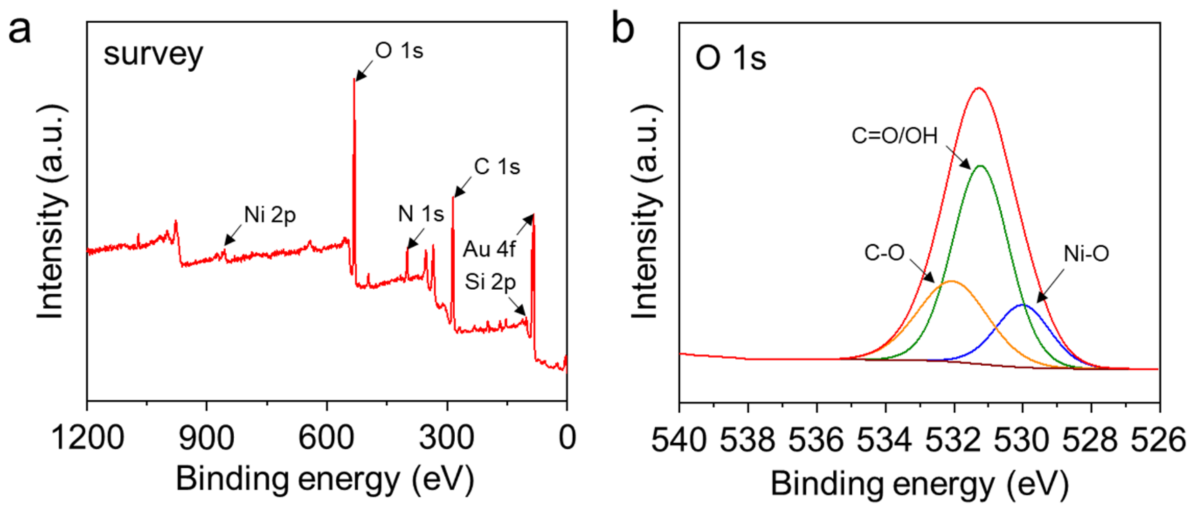

Figure S7. Surface chemical composition analysis of Au-HA@Ni-MOF/NF. (a) XPS survey spectrum. (b) High-resolution O 1s XPS spectra.

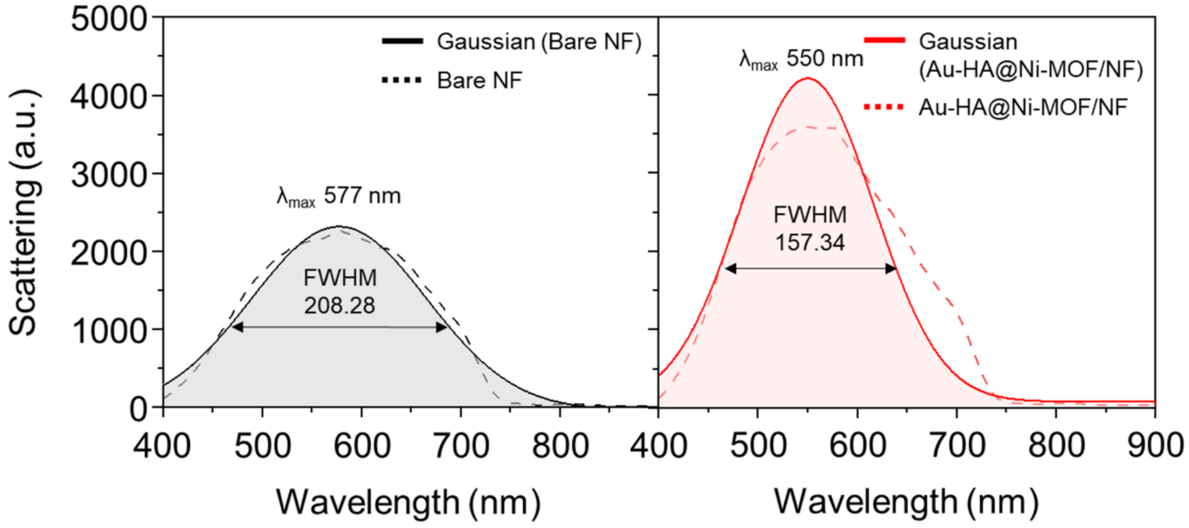

Figure S8. Approximating Gaussian distribution using each scattering data. The exhibition for full width at half maximum (FWHM) and maximum peak (λ_{max}) in a Gaussian distribution.

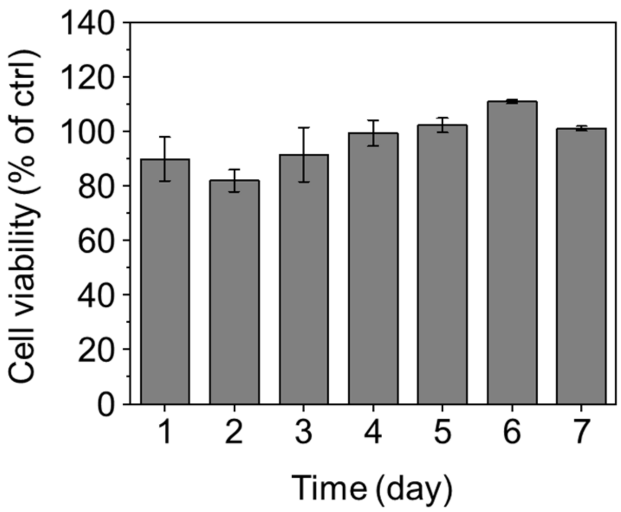

Figure S9. Assessment of long-term (1 week) cell viability on Au-HA@Ni-MOF evaluated by CCK-8 assay.

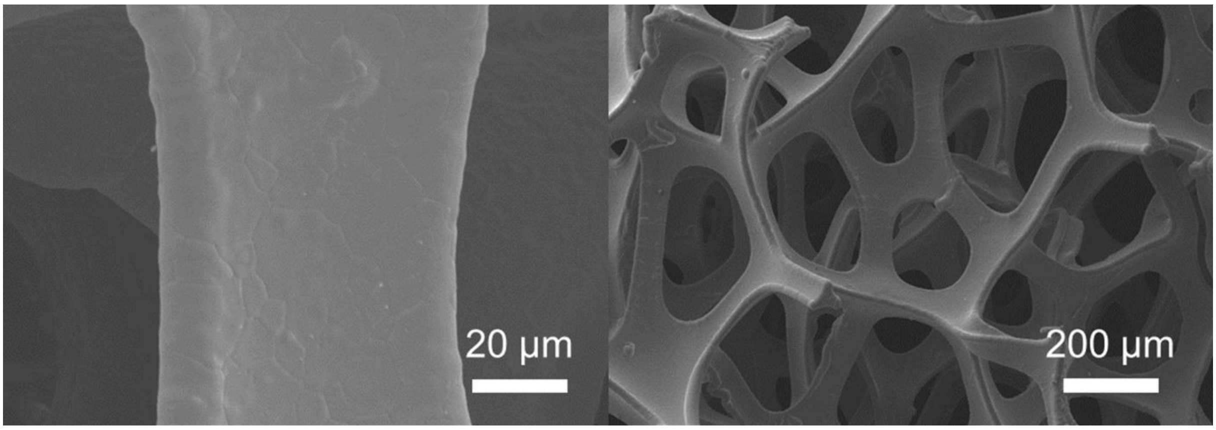

Figure S10. Low magnification FE-SEM images of the 3D structure of bare Ni foam.

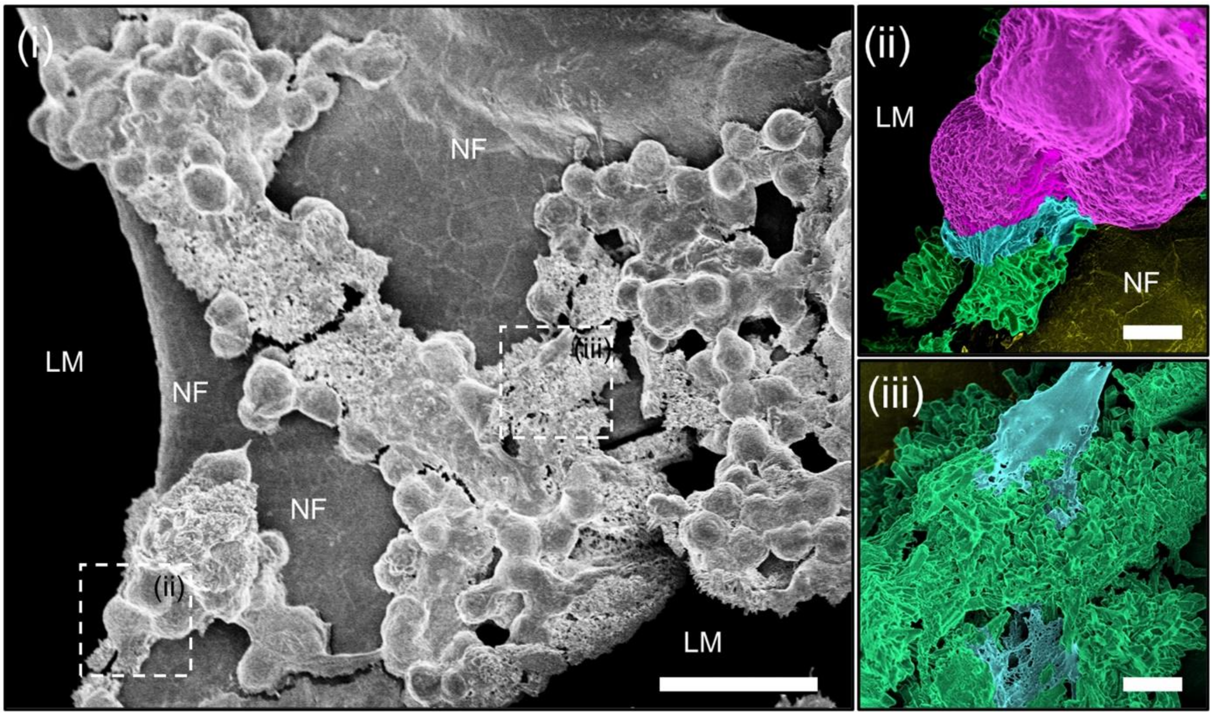

Figure S11. SEM images of the alveoli-like hollow 3D scaffold. (i) Low magnification image of Au-HA@Ni-MOF/NF backbone (scale bar: 40 µm). (ii) Magnified and color-coded sectional images showing the cells aggregated on the Au-HA@Ni-MOF/NF (scale bar: 5 µm). (iii) Ni-MOF structure (emerald green) on NF. Abbreviations: Lumen (LM), and nickel foam backbone (NF).

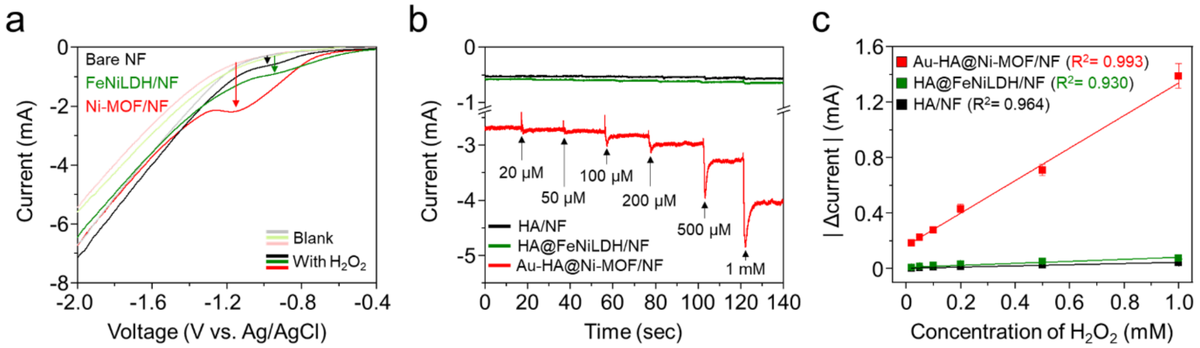

Figure S12. Comparison of electrochemical performance of the scaffolds. (a) LSV measurement of bare NF, FeNiLDH/NF, and Ni-MOF/NF with/without 10 mM H_2O_2 in 0.1 M PBS. (b) Amperometric transient current density vs. time plot of HA/NF, HA@FeNiLDH/NF, and Au-HA@Ni-MOF/NF under H_2O_2 injection of different concentrations from 100 nM to 1 mM. (c) Calibration curve of H_2O_2 concentration vs. |Δ current| for the HA/NF, HA@FeNiLDH/NF, and Au-HA@Ni-MOF/NF.

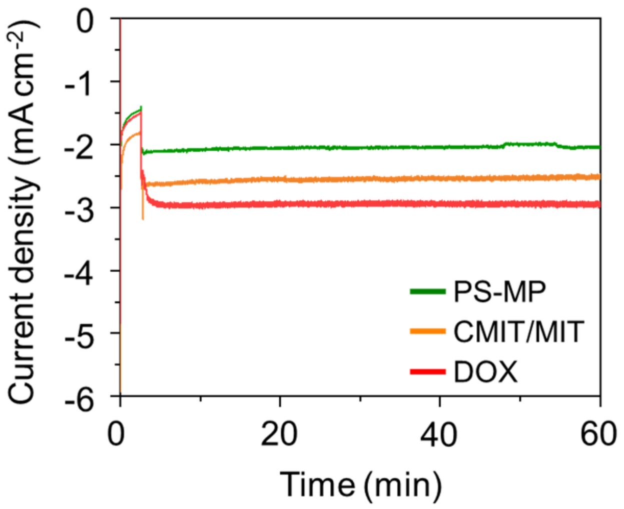

Figure S13. Stability of the electrochemical detection: chronoamperometry test of A549 cell-cultured Au-HA@Ni-MOF/NF at a constant potential of -1.17 V (vs. Ag/AgCl) for 60 min, with the injection of different toxicants (PS-MPs, CMIT/MIT, and DOX).

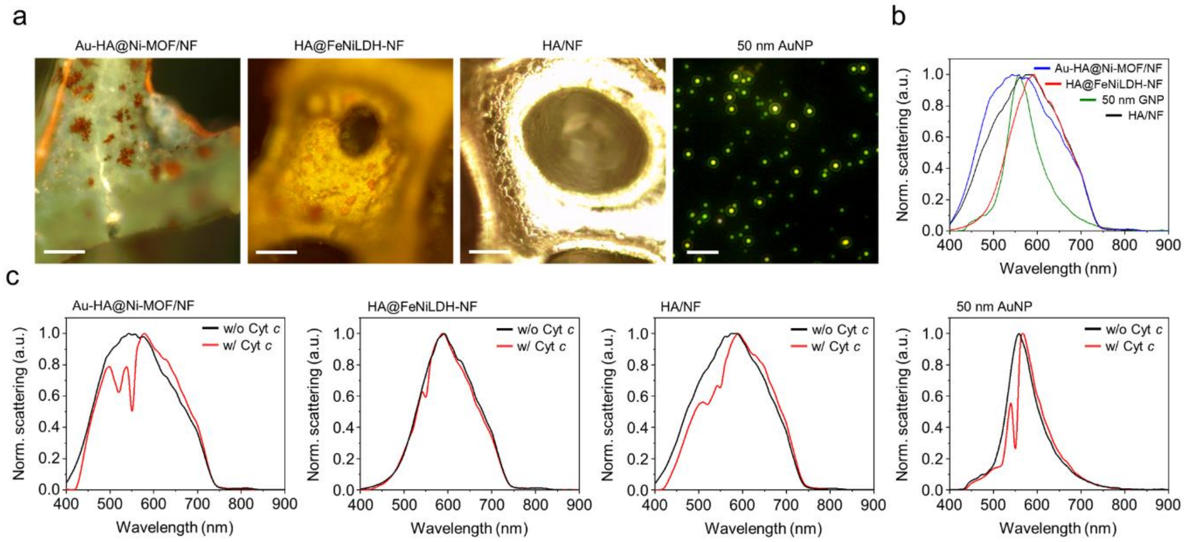

Figure S14. Comparison of Cyt *c*–mediated PRET signals of Au-HA@Ni-MOF/NF and other comparative substrates. (a) Representative dark-field scattering images of HA/NF, Au-HA@Ni-MOF/NF, HA@FeNiLDH/NF and 50 nm gold nanoparticles (AuNP). (b) Rayleigh scattering spectra of the tested three substrates (scale bar: 50 μm) and single AuNP (scale bar: 10 μm). (c) Quenching dip changes in the scattering spectra of each substrate induced by reduced Cyt *c*.

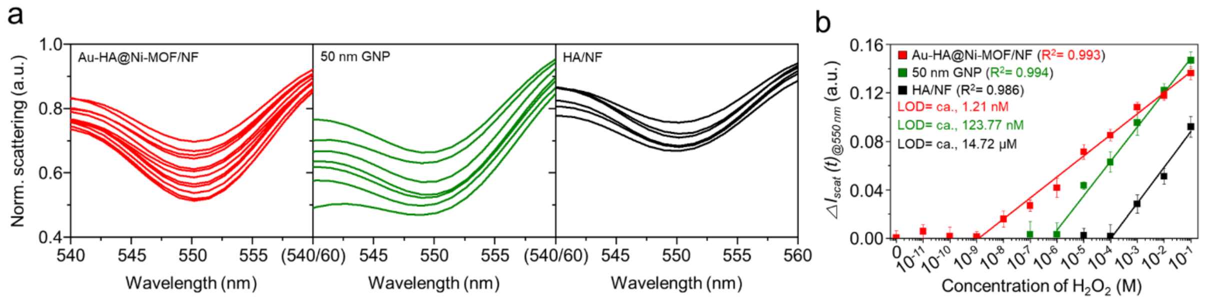

Figure S15. Quantification of PRET-based optical detection performance of the substrates. (a) H_2O_2 concentration-dependent changes in the spectral quenching dip of each substrate. (b) Calibration curves for H_2O_2 obtained with Au-HA@Ni-MOF/NF, HA/NF and 50 nm AuNP.
